# The Influence of Social Media on the Body Image of First Year Female Medical Students of University of Khartoum, 2022

**DOI:** 10.1192/bjo.2024.141

**Published:** 2024-08-01

**Authors:** Razan Farah

**Affiliations:** University of Khartoum, Faculty of Medicine, Khartoum, Sudan

## Abstract

**Aims:**

Facebook, Instagram, TikTok and other social media applications have become an integral component of everyone's social life, particularly among younger generations and adolescents. These social apps have been changing a lot of conceptions and beliefs in the population by representing public figures and celebrities as role models. The social comparison theory, which says that people self-evaluate based on comparisons with similar others, is commonly used to explore the impact of social media on body image. There is a need to study the influence of those social platforms on the body image as there has been an increase in body dissatisfaction in the recent years.

**Methods:**

This was a cross sectional study that used a self administered questionnaire on a simple random sample of 133 female medical students of the first year. Data were analyzed using SPSS.

**Results:**

Finding shows that the response rate was 75%. There was an association between social media usage and noticing how the person looks (p value = 0.022), but no significant association between social media use and body image influence or dissatisfaction was found.

**Conclusion:**

This study implies more research under this topic in Sudan as the literature are scarce.

